# *In Vitro* Antidiabetic Activity and Mechanism of Action of* Brachylaena elliptica* (Thunb.) DC

**DOI:** 10.1155/2018/4170372

**Published:** 2018-07-04

**Authors:** Idowu Jonas Sagbo, Maryna van de Venter, Trevor Koekemoer, Graeme Bradley

**Affiliations:** ^1^Plant Stress Group, Department of Biochemistry and Microbiology, University of Fort Hare, P.O. Box X1314, Alice, South Africa; ^2^Department of Biochemistry and Microbiology, Nelson Mandela Metropolitan University, P.O. Box 77000, Port Elizabeth 6031, South Africa

## Abstract

In South Africa, the number of people suffering from diabetes is believed to be rising steadily and the current antidiabetic therapies are frequently reported to have adverse side effects. Ethnomedicinal plant use has shown promise for the development of cheaper, cost-effective antidiabetic agents with fewer side effects. The aim of this study was to investigate the antidiabetic activity and mechanism of action of aqueous leaf extract prepared from* Brachylaena elliptica*. The potential of the extract for cytotoxicity was evaluated using MTT assay in HepG2 cells. The effects of the plant extract on glucose utilization in HepG2 cells and L6 myotubes, triglyceride accumulation in 3T3-L1, INS-1 proliferation, glucose metabolism in INS-1 cells, and NO production in RAW macrophages were also investigated using cell culture procedures. The inhibitory effects of the extract on the activities of different enzymes including alpha-amylase, alpha-glucosidase, pancreatic lipase, dipeptidyl peptidase IV (DPP-IV), collagenase, and CYP3A4 enzymes were evaluated. The extract also tested against protein glycation using standard published procedure. The plant extract displayed low level of toxicity, where both concentrations tested did not induce 50% cell death. The extract caused a significant increase in glucose uptake in HepG2 liver cells, with efficacy significantly higher than the positive control, berberine. The crude extract also displayed no significant effect on muscle glucose uptake, triglyceride accumulation in 3T3-L1, glucose metabolism in INS-1 cells, alpha-amylase, alpha-glucosidase, DPP-IV, lipase, protein glycation, and collagenase compared to the respective positive controls. The extract displayed a proliferative effect on INS-1 cells at 25 *μ*g/ml when compared to the negative control. The plant also produced a concentration-dependent reduction in NO production in RAW macrophages and also demonstrated weak significant inhibition on CYP3A4 activity. The findings provide evidence that* B. elliptica* possess antidiabetic activity and appear to exact its hypoglycemic effect independent of insulin.

## 1. Introduction

Diabetes mellitus is a serious complex multifactorial disorder characterized by hyperglycemia (very high blood glucose level) and glucose intolerance, either due to the relative deficiency in insulin secretion or impaired the effectiveness of insulin's action to enhance glucose uptake. If left untreated, it can lead to severe complications. These complications include hyperlipidemia (abnormal high level of lipid in the blood), oxidative stress, and enzymatic glycation of protein [1]. Considering the fact that diabetes is regarded as a chronic metabolic disease, numerous antidiabetic therapies with conventional drugs are often not a single-dose program as most drugs require frequent injections, sometimes for the entire life of the diabetic patient. However, many of these conventional drugs have been reported for their inefficiency with prominent adverse side effects [2]. These limitations have largely prompted the exploration of management strategies involving the use of medicinal plants reported to be cost-effective antidiabetic agents with fewer reported side effects [3]. However, the majority of these traditional plants have not been scientifically validated for their efficacy in the treatment of diabetes.* B. elliptica* is among such plants traditionally used among local healers in the Eastern Cape province of South Africa [4]. Yet no scientific research has confirmed the efficacy of this plant with regard to diabetes mellitus. Therefore, determination of its efficacy is very important as this plant may play a significant role in the management of diabetes mellitus.

*Brachylaena elliptica* (family Asteraceae) is commonly known as isiduti (Xhosa); iphahle and uhlunguhlungu (Zulu); and Bitterblaar or suurbos (Afrikaans). It is a small tree (4 m tall), with a light grey to brown bark that becomes rough with age. The plant is dispersed from Port Elizabeth, Eastern Cape Province to Durban, KwaZulu-Natal Province. The leaves are lanceolate, elliptic to ovate, dim green above and white felted beneath. The species occurs in the bushveld on rough outcrops and alongside the edge of the evergreen forest. Poles from this species are utilized as fence posts; the sticks have been purportedly used to start a fire by friction. The leaves, which are intensely bitter tasting, are reportedly used traditionally [5] and valued by the Zulu and Xhosa for the management of diabetes. The infusion of the leaves is used as a gargle and mouthwash [6]. Previously we have demonstrated the antioxidant, phytochemical, and antibacterial activities of* B. elliptica* leaf extract [7]. The present study was therefore undertaken to investigate the antidiabetic activity and mechanism of action using various* in vitro* models designed to stimulate specific antidiabetic targets.

## 2. Materials and Methods

### 2.1. Collection of Plant Materials

The leaves of* B. elliptica* were collected from a thick forest in Amathole District in Eastern Cape. The plant was identified by its vernacular name and later confirmed at the Department of Botany, University of Fort Hare, by Professor D.S. Grierson and Voucher specimen with corresponding number BRA (47) 8936 was deposited at the Griffin Herbarium of the University of Fort Hare.

### 2.2. Preparation of Extracts

The leaves were oven dried to constant weight at 40°C and further pulverized to a homogeneous powder using an electric blender (Waring Products Division, Torrington, USA). Sixty (60) grams of the powdered plant was extracted in 1000 ml of distilled water. The extract was then filtered using a Buchner funnel and Whatman No. 1 filter paper. The extract was immediately frozen at -40°C and dried for 48 h using a freeze dryer to give a yield of 9 g. The dried extract was stored at -4°C and later reconstituted in DMSO just before various bioactivity determinations.

### 2.3. Cell Lines, Media, Reagents, and Assay Kits

The HepG2 liver cells, INS-1 cells, L6 myoblasts, RAW 264.7 macrophages cells, and 3T3-L1 cells were obtained from Highveld Biological, South Africa. The Eagle's minimum essential medium (EMEM), MTT (3-(4, 5-dimethylthiazol-2yl)-2, and 5-diphenyl tetrazolium bromide were obtained from Sigma Aldrich, South Africa. The fetal calf serum (FCS) and phosphate-buffered saline (PBS) were obtained from Lonza Biologics. All other reagents used in this study were of analytical grade and purchased from Sigma or Merck Chemicals.

### 2.4. Maintenance of Cell Cultures

All cell cultures were incubated at 37°C in a humidified atmosphere with 5% CO_2_. The HepG2 cells were replenished with growth medium every 2-3 days, consisting of RPMI 1640 medium supplemented with 10% fetal calf serum. The L6 myoblasts cells were cultured in antibiotic-free growth medium consisting of RPMI 1640 supplemented with 10% fetal calf serum. 3T3-L1 cells were cultured in DMEM with 10% fetal bovine serum. The INS-1 cells were cultured in RPMI containing 5% fetal calf serum. The RAW 264.7 macrophages cells were cultured in Dulbecco's modified Eagle's medium (DMEM) containing l-glutamine supplemented with 10% FCS and 1% PSF solution. All cell lines were subcultured after 90% confluence was reached. The 3T3-L1 cells were cultured in DMEM with 10% fetal bovine serum.

### 2.5. Cytotoxicity Assay

The cytotoxicity assay was determined according to the method described by Mosmann [8] with slight modification. The HepG2 liver cells were seeded into 96-well plates at a density of 8000 cells per well within a volume of 100 *μ*l. The cells were left to attach overnight and then treated with 100 *μ*l of plant extract at various concentrations (50 *μ*g/ml, 100 *μ*g/ml, and 200 *μ*g/ml) to the specific well. After 48 h of incubation at 37°C, the spent medium was initially removed by aspiration from the cells and 100 *μ*l of EMEM medium containing 10% FCS and 0.5 mg/ml MTT (dissolve 25 mg MTT in 50 ml complete culture medium) was added and further incubated for 3 h at 37°C. The medium was later aspirated and MTT crystal (purple formazan) was then dissolved in DMSO (200 *μ*l/well) to solubilize the formazan crystals formed in the cells. The absorbance was read at 540 nm using a microplate reader (Multiscan MS, Labsystem). The cytotoxicity of the plant extract was expressed as percentage of control (medium only), which was taken as zero and IC_50_ values were calculated.

(1) %  Cell  death=1−Absorbance  of  test  wellAverage  of  the  untreated×100

### 2.6. Glucose Utilization Experimental Procedure on HepG2

The glucose utilization in HepG2 cells was determined by the method described by van de Venter* et al*. [9]. The HepG2 cells were dislodged by brief exposure to 0.25% Trypsin in phosphate-buffered saline, counted, suspended in new growth medium, and then seeded at a density of 6 000 cells per well into a 96-well culture plate (Nunc, Denmark) and allowed to adhere and grow in a humidified incubator with 5% CO_2_ at 37°C for three days. Two cell-free rows were also included to serve as blanks. On day three after seeding, without changing the medium, 10 *μ*l of the plant extract at a concentration dose of 25 *μ*g/ml and 100 *μ*g/ml was added to each well. After 48 h incubation, the spent culture medium was removed by aspiration and replaced with a 25 *μ*l incubation buffer (RPMI medium diluted with PBS, 0.1% BSA and 8 mm glucose) and further incubated for an additional 3 h at 37°C. Metformin (0.1 *μ*g/ml) and berberine (18 *μ*g/ml) were used as the positive controls while the negative control (untreated) contained only the incubation buffer without extract. After incubation, 10 *μ*l of the incubation medium was removed from each well and transferred into a new 96-well plate into which 200 *μ*l of glucose oxidase reagent (SERA-PAK Plus, Bayer) was added to determine the concentration of glucose in the medium. After 15 min of incubation at 37°C, the absorbance was measured at 492 nm using a Multiscan MS microtitre plate reader (Lab Systems). The amount of glucose utilized was calculated as the difference between the cell-free and cell-containing wells. The percentage of glucose utilization was calculated in relation to the untreated controls. Cell viability in the representative well was determined using the MTT assay [8].

### 2.7. Glucose Utilization Experimental Procedure on L6 Myoblasts

The glucose utilization in L6 myoblasts cells was determined according to the methods described by van de Venter* et al*. [9]. The L6 cells were seeded into 96-well culture plates at a density of 3 000 cells/well and allowed to adhere until 90% confluence was reached. Two cell-free rows were also included to serve as blanks for glucose utilization assay. After 90% confluence, the culture medium was removed and replaced with DMEM containing 2% FBS and cultured for an additional five days. Fourty-eight hours (48 h) prior to the glucose utilization assay, the culture medium was replaced and 10 *μ*l of plant extract at various concentrations of 12.5 *μ*g/ml, 25*μ*g/ml, and 50 *μ*g/ml was added to separate wells. A column was also treated with insulin (4 *μ*g/ml) instead of the plant extract to serve as a positive control. The cells were incubated in the presence of the extract for an additional 48 h. After the incubation period, the spent medium was removed and replaced with a 25 *μ*l incubation buffer containing RPMI medium diluted with PBS, 0.1% BSA, and 8mM glucose and incubated for further 3 h at 37°C. Five microlitres (5*μ*l) of the incubation medium was removed from each well and then placed into a new 96-well plate into which 200 *μ*l of glucose oxidase reagent (SERA-PAK Plus, Bayer) was added per well to determine the concentration of glucose in the medium. After 15 min of incubation at 37°C, the absorbance was measured at 520 nm using a Multiscan MS microtitre plate reader (Lab Systems). The amount of glucose utilized was calculated as the difference between the cell-free and cell-containing wells. The percentage of glucose uptake was calculated in relation to the untreated controls. Cell viability in the representative well was determined using the MTT assay [8].

### 2.8. Lipid Accumulation in 3T3-L1 Preadipocytes

Lipid accumulation in 3T3-L1 preadipocytes cells was determined according to the method described by Oyedemi* et al.* [10]. The 3T3-L1 preadipocytes were seeded at a density of 6 000 cells per well into a 48-well culture plate (Nunc, Denmark) and allowed to grow until 100% confluence was reached. Two days after confluence preadipocytes were treated for an additional two days with various concentrations (prepared at 12.5 *μ*g/ml, 25 *μ*g/ml, and 50 *μ*g/ml) of the plant extract or positive control (Rosiglitazone; 0.4 *μ*g/ml). The cells were then cultured for an additional ten days in normal culture medium (DMEM with 10% FBS) and the medium replaced every two to three days. After ten days, the spent culture medium was removed and gently washed with PBS. The cells were then allowed to fix at room temperature for approximately 1 h by adding 500 *μ*l per well of 10% formaldehyde in PBS. The fix solution was aspirated and later stained by adding 200 *μ*l of prewarmed oil red working solution (6 ml of stock solution (0.5 g oil red dye in 100 ml isopropanol) in 4ml of distilled water) for 15 min at 37°C. After 15 min of incubation, excess dye was extensively washed with water and the plate dried in an oven at 37°C. The dye was further extracted by adding isopropanol (250 *μ*l per well) after which 200 *μ*l was transferred to a 96-well plate and the absorbance measured at 520 nm using a Multiscan MS microtitre plate reader (Lab Systems).

### 2.9. Glucose Metabolism as a Reflection of Insulin Secretion

The glucose metabolism assay as a reflection of insulin secretion in INS-1 cells using MTT (tetrazolium) colorimetric assay was determined according to the method described by Janjic and Wollheim [11]. The INS-1 cells were cultured in RPMI containing 5% FCS. The cells were seeded into 96-well plates at a density of 8000 cells per well, with a volume of 100 *μ*l. The cells were left to attach overnight and treated with plant extract (100 *μ*g/ml) or PBS (which serve as a control) in the presence or absence of glucose (20 mM). After 48 h of incubation at 37°C, the spent medium was removed from the cells and 100 *μ*l of EMEM medium containing 10% FCS and 0.5 mg/ml MTT was added and further incubated for additional 30 min at 37°C. The medium was later aspirated and MTT crystal (purple formazan) dissolved in DMSO (200 *μ*l/well). The absorbance was read at 540 nm using a microplate reader (Multiscan MS, Lab Systems).

### 2.10. INS-1 Proliferation Assay Using ImageXpress® Micro XLS Analysis

The cell proliferation assay was carried out using a method described by Sirenko et al. [12] with some modifications. Rat pancreatic insulinomas (INS-1) cells were cultured in RPMI containing 5% FCS, seeded into 96-well plates at a density of 8000 cells per well, with a volume of 100 *μ*l. The cells were left for 16 h to attach overnight and then treated by adding 100 *μ*l of the plant extract at various concentrations (prepared at 12.5 *μ*g/ml, 25*μ*g/ml, and 50 *μ*g/ml). A well was also treated with gamma-aminobutyric acid (GABA; 10 *μ*g/ml) instead of the plant extract to serve as a positive control while DMSO (0.25%, v/v) served as the vehicle (untreated) control. The cells were then stained with 10 *μ*l of the Hoechst staining solution (10 *μ*g/ml in PBS) for 10 min. After 10 min, the staining solution was removed and 10 *μ*l of propidium iodide (PI) solution (10 *μ*g/ml in PBS) was added and incubated for 10 min. The cells were then observed under ImageXpress Micro (Molecular Devices) automated epifluorescent microscope. The images were later acquired and analyzed using a Multiwavelength Cell Scoring application module to calculate the percentage of cells positive for each dye (Hoechst 33342 for total cell counts and propidium iodide for dead cell counts).

### 2.11. ImageXpress Micro (Molecular Devices) Acquisition

Images were taken with a Molecular Devices ImageXpress Micro XLS microscope using the blue and red filters, as well as phase contrast with 40X objective. Nine fields were acquired per well and cells were scored by defined dimensions, analyzed by the MetaXpress software.

### 2.12. Inhibition of Nitric Oxide Production in RAW Macrophage Cells

Inhibition of nitric oxide production in RAW macrophages was determined according to the method described by Yang* et al*. [13] with slight modification. The RAW macrophage cells were seeded into a 96-well culture plate at a density of 5 000 cells per well and allowed to attach overnight. The spent culture medium was removed and replaced with fresh medium containing different concentrations (12.5 *μ*g/ml, 25 *μ*g/ml, 50 *μ*g/ml, and 100 *μ*g/ml) of the extract to give a total volume of 50 *μ*l per well. Aminoguanidine (4 *μ*g/ml) was used in place of the extract, as a positive control. Fifty microlitres (50 *μ*l) of lipopolysaccharides (LPS) containing medium (100 *μ*g/ml) was added to all the wells except for those of the blank. After 18 h of incubation, 50 *μ*l of the medium was removed and transferred into a new 96 well plate into which 50 *μ*l of Griess reagents (1:1 mixture, v/v of 1% sulfanilamide and 0.1% naphthylethylenediamine dihydrochloride in 5% phosphoric acid) was added and incubated for 11 min. Then the absorbance was measured at 540 nm using a Multiscan MS microtitre plate reader (Lab Systems). The toxicity test by Mosmann [8] was performed on the remaining cells by adding 100 *μ*l MTT (3-(4,5-dimethylthiazol-2-yl)-2,5-diphenyltetrazolium bromide) solution to obtain a final concentration of 0.5 mg/ml. After 15 min incubation, the spent culture medium was removed and 100 *μ*l of DMSO was added to solubilize the MTT crystal. The absorbance was then measured at 540 nm using a Multiscan MS microtitre plate reader (Lab Systems).

### 2.13. Alpha-Amylase Inhibition Assay

The alpha-amylase assay was performed according to the method described by Odeyemi [14]. Briefly, 15 *μ*l of the plant extract at different concentrations (50 *μ*g/ml – 200 *μ*g/ml) (diluted in a phosphate buffer) was added to 5 *μ*l of enzyme porcine pancreatic solution into 96-well plate. After 10 min of incubation at 37°C, the reaction was initiated by adding 20 *μ*l of starch solution and further incubated for 30 min at 37°C. The reaction was then stopped by adding 10 *μ*l 1M of HCl to each well followed by 75 *μ*l of iodine reagent. A blank containing phosphate buffer (pH 6.9) instead of the extract and a positive control (acarbose, 64 *μ*g/ml) were prepared. No enzyme control and no starch control were included for each test sample. The absorbance was measured at 580 nm and the percentage inhibitory activity was calculated by using the following equation:

(2) %  Inhibition=1−Absorbance  of  the  untreated  (Control)Absorbance  of  the  test  well×100

### 2.14. Alpha-Glucosidase Inhibition Assay

The alpha-glucosidase inhibition assay was determined using a method described by Sancheti* et al*. [15] with slight modification. Briefly, 5 *μ*l of the plant extract (prepared at concentration of 50 *μ*g/ml, 100 *μ*g/ml, and 200 *μ*g/ml) was added to 20 *μ*l of 50 *μ*g/ml alpha-glucosidase solution into a well of a 96-well plate. Thereafter, 60 *μ*l of 67 mM potassium phosphate buffer (pH 6.8) was then added. After 5 min of incubation, 10 *μ*l of 10 mM *ρ*-nitrophenyl-*α*-D-glucoside solution (PNP-GLUC) was then added and further incubated for 20 min at 37°C. After incubation, 25 *μ*l of 100 mM Na_2_CO_3_ (sodium carbonate) solution was added and the absorbance was measured at 405 nm. A blank and sample blank were also prepared by adding 5 *μ*l of deionised water instead of plant extract and 20 *μ*l of deionised water instead of enzyme, respectively. Epigallocatechin gallate (10 *μ*g/ml) was used as a positive control. The percentage inhibition was calculated using the following equation:

(3) %  Inhibition=1−Absorbance  of  the  test  wellAbsorbance  of  the  untreated  (control)×100

### 2.15. Lipase Inhibition Assay

The lipase inhibition assay was determined according to the method described by Lewis and Liu [16]. Briefly, 10 *μ*l of the plant extract or positive (orlistat; 50 *μ*g/ml) or negative control (distilled water) was added to the well of 96-well plates. Thereafter, porcine pancreatic solution (10 mg/ml) was freshly prepared in 50 mM Tris-HCl buffer (pH 8.0) and centrifuged to remove insoluble material and was then added at 4 times the volume to each of the sample (40 *μ*l). After 15 min of incubation, 170 *μ*l of substrate solution (20 mg pNPP in 2ml isopropanol added to 18 ml 50 Mm Tris-HCl buffer (pH 8.0) containing 20 mg gum Arabic, 40 mg sodium deoxycholate, and 100 *μ*l Triton X-100) was then added and incubated for 25 min at 37°C; the absorbance was then measured at 405 nm using a Biotek® PowerWave XS spectrophotometer and the percentage inhibition was calculated using the following equation:

(4) %  Inhibition=1−Absorbance  of  the  test  wellAbsorbance of  the  untreated  (control)×100

### 2.16. DPP-IV Inhibition Assay

The DPP-IV inhibition assay was carried out according to the method described by Al-masri* et al*. [17] with slight modification. Briefly, 35 *μ*l of the plant extract (50 *μ*g/ml – 100 *μ*g/ml) or positive control (diprotin A; 50 *μ*g/ml) was added to 15 *μ*l of human recombinant DPP-IV enzyme solution (50 *μ*U/*μ*l in Tris buffer) in respective wells of a 96-well plate. After 5 min of incubation at 37°C, 50 *μ*l of 20 mM *ρ*NA substrate (Gly-Pro- *ρ*NA) dissolved in Tris buffer was added to initiate the reaction and further incubated for 30 min at 37°C. After incubation, 25 *μ*l of 25% acetic acid solution was added to stop the reaction and the absorbance was measured at 410 nm. A blank and sample blank were also prepared by adding 35 *μ*l of buffer instead of plant extract and 15 *μ*l of buffer instead of enzyme, respectively. The percentage inhibition was calculated using the following equation:

(5) %  Inhibition=1−Absorbance  of  the  test  wellAbsorbance  of  the  untreated  (control)×100

### 2.17. Protein Glycation Assay

Briefly, 50 *μ*l of protein solution (100 mg gelatine in 5ml of distilled water) was added to 10*μ*l of glyceraldehyde solution (222 mg in 5ml distilled water) in black microplates. The plate was then sealed and incubated at 37°C for 24 h. After incubation, 40 *μ*l of the plant extract (prepared at concentrations of 50 *μ*g/ml and 100 *μ*g/ml) was added. A blank containing distilled water instead of the extract served as a negative control (untreated) while aminoguanidine (100 *μ*M) was used as a positive control. The fluorescence was then measured at 370 nm (excitation); 440 nm (emission). The experiments were performed in triplicate and the percentage inhibition was then calculated using the following formula:

(6) %  Inhibition=1−Fluorescence  of  test  wellFluorescence  of  negative  control×100

### 2.18. Collagenase Inhibition Assay

Briefly, 10 *μ*l of enzyme was added to 10*μ*l of the plant extract (prepared at concentrations of 12.5 *μ*g/ml, 25 *μ*g/ml, and 50 *μ*g/ml) or positive control (EDTA; 6 *μ*g/ml), gelatine (2 mg/ml), and buffer (50 mM Tris-HCl buffer (pH 7.4)). The resulting mixture was then incubated for 1 h at 37°C. After incubation, 20 *μ*l of Coomassie brilliant blue (CBB) was added and then centrifuged at 500 rcf for 5 min. After the supernatant was removed, 50 *μ*l of washing solution (40% methanol/ 10% acetic acid) was added to wash the pellet to remove excess CBB and then dissolved in 50 *μ*l of DMSO. The absorbance was measured at 540 nm using a Multiscan MS microtitre plate reader (Lab Systems). The experiments were performed in triplicate and the percentage inhibition was then calculated using the following formula:

(7) %  Inhibition=1−Absorbance  of  the  untreated  (Control)Absorbance  of  the  test  well×100

### 2.19. Cytochrome P450 (3A4) Inhibition Assay

The effects of the plant extract were screened against recombinant human CYP3A4 enzyme activity using Vivid CYP assay kits (BOMR substrate CYP3A4 red) according to the manufacturer's instructions. The kit contained vivid CYP450 reaction buffer, CYP450 BACULOSOMES reagent, vivid fluorescent substrate, vivid fluorescent standard, the vivid regeneration system (Part no. P2878; 333 mM glucose-6-phosphate and 30 U/mL glucose-6-phosphate dehydrogenase in 100 mM potassium phosphate, pH 8.0), and 0.5 ml vivid NADP^+^ (Part no. P2879; 10 mM NADP^+^ in 100 mM potassium phosphate, pH 8.0). The vivid regeneration system and vivid NADP^+^ were stored at -80°C.

### 2.20. Preparation of Stock

All the reactant mixtures were allowed to thaw for 10–15 min. The vivid substrates were reconstituted using anhydrous acetonitrile and fluorescent standards were reconstituted using DMSO and DMSO/water (1:1), respectively.

### 2.21. Reaction Procedure

Briefly, 40 *μ*l of the 2.5x of the plant extract or positive control (ketoconazole, 230 *μ*M and 92 *μ*M) was added to the respective wells of the black 96-well plates. 50 *μ*l of the master pre-mix (P450 Baculosomes Plus reagent and vivid regeneration system in 1X vivid CYP450 Reaction Buffer) was then added to each well and incubated for 10 min at room temperature to allow the compound or ketoconazole to interact with the CYP3A4 enzyme. After incubation, the reaction was then initiated by adding 10X substrate (10 *μ*l) consisting of BOMR substrate and NADP^+^ diluted in reaction buffer. The reaction mixture was incubated for 30 min at room temperature. The reaction was stopped by the addition of 50 *μ*l of 0.5 M Tris base. The fluorescence was measured at 550 nm (excitation) and 590nm (emission) using a Biotek Synergy MX fluorimeter. The same procedure was applied for the standards. A solvent control was also prepared; this contained only the reaction buffer instead of the sample. The percentage inhibition was calculated using the following formula:

(8) $$\begin{array}{c} \%\text{ Inhibition}=\left(\;1-\frac{C-B}{A-B}\;\right)\times 100 \end{array}$$

where C is the fluorescence intensity observed in the presence of the sample, A is the fluorescence observed in the solvent control, and B is the fluorescence intensity observed in the presence of the positive inhibition control.

### 2.22. Statistical Analysis

For the INS-1 proliferation assay, statistical analysis was carried out using Graph Pad Prism Version 5.01 and the test of significance was done using Student's t-test (two-tailed). Replicate values for each treatment were compared with replicate values of the control wells. Level of significance ranged from p<0.001 to p<0.05.

For all the experiments except INS-1 proliferation, statistical analysis was carried out with a one-way analysis of variance (ANOVA) and the difference between samples was determined by Duncan's Multiple Range test using the Minitab program (12.11.1). The data were expressed as the mean ± standard deviation and values were considered significant at p < 0.05.

## 3. Results

### 3.1. Cytotoxicity

**Figure 1:**
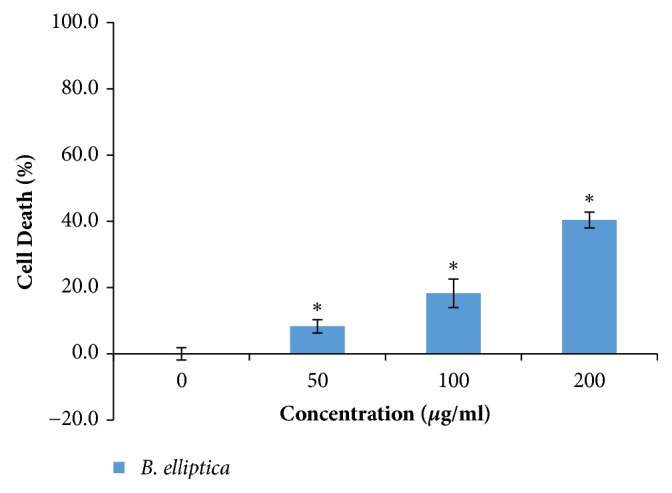
MTT cytotoxicity effect of the aqueous extract of* B. elliptica* in HepG2 liver cells. Data are expressed as % of control ± SD (n = 4). *∗* indicates a significant increase relative to the untreated control (p < 0.05).

The* in vitro* cytotoxic activity of the extract of* B. elliptica* was measured by the MTT assay against the HepG2 liver cell line at various concentrations. The cytotoxicity result revealed that* B. elliptica* extract displayed low level of toxicity to HepG2 cells at all the doses tested in a dose-dependent manner (Figure 1). However, at the highest dosage (200 *μ*g/ml) tested, the extract showed less than 50% cell death, whereas the IC_50_ value (concentration that can cause 50 % cell death) was calculated to be 250 *μ*g/ml.

### 3.2. Glucose Utilization in HepG2

**Figure 2:**
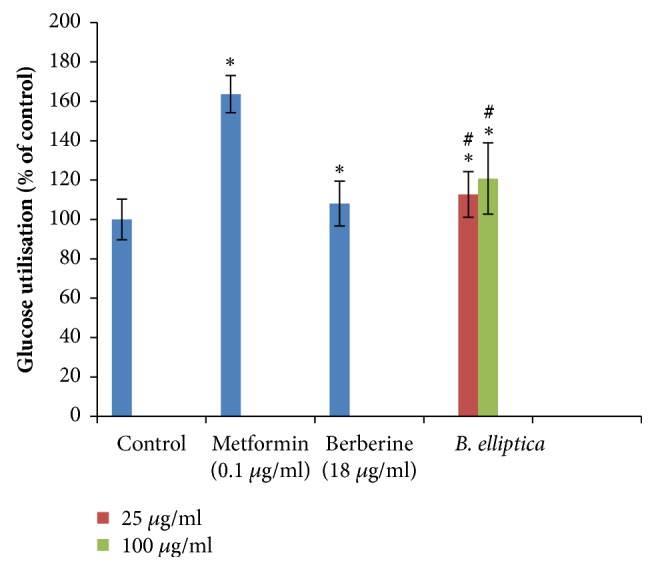
Effect of* B. elliptica* extract on glucose utilization in HepG2 hepatocytes. Cells were treated for 48 h in the presence or absence of varying concentration of the plant extract. Data expressed as mean ± SD (n = 4). *∗* indicates a significant increase relative to the untreated control (p < 0.05). (#) indicates a significant increase relative to the positive control (berberine) (p < 0.05). No significant increase relative to the metformin (positive control) was noted.

The results obtained for glucose uptake in HepG2 cells in the presence of the plant extract at 25 and 100 *μ*g/ml are presented in Figure 2. The crude extract of* B. elliptica* caused a significant (p < 0.05) higher increase in glucose uptake in HepG2 cells at all the concentration tested in a concentration-dependent manner when compared to the untreated control and berberine, with the exception of metformin. On the other hand, the crude extract of* B. elliptica* (121%) also exhibited higher increase in glucose uptake at 100 *μ*g/ml when compared to berberine (108%) but lower than that observed for metformin (164%).

**Figure 3:**
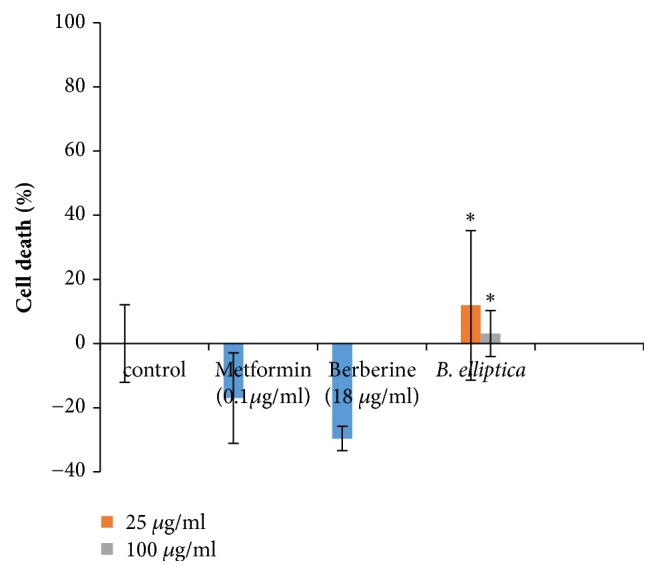
Toxicity of* B. elliptica* extract to HepG2 cells used for glucose uptake assay. Data represent the mean ± SD (*n* = 4). *∗* indicates a significant increase relative to the untreated control (p < 0.05).

The toxicity assay revealed that* B. elliptica *extract was not toxic to HepG2 cells, producing less than 10% cell death at both concentrations investigated (Figure 3). However, berberine and metformin displayed no significant toxicity but rather proliferated the cells. Furthermore, the low level of cell death exhibited by this extract and the positive controls most likely explains the significant reduction in glucose uptake in the cells.

### 3.3. Glucose Utilization in L6 Myoblast

**Figure 4:**
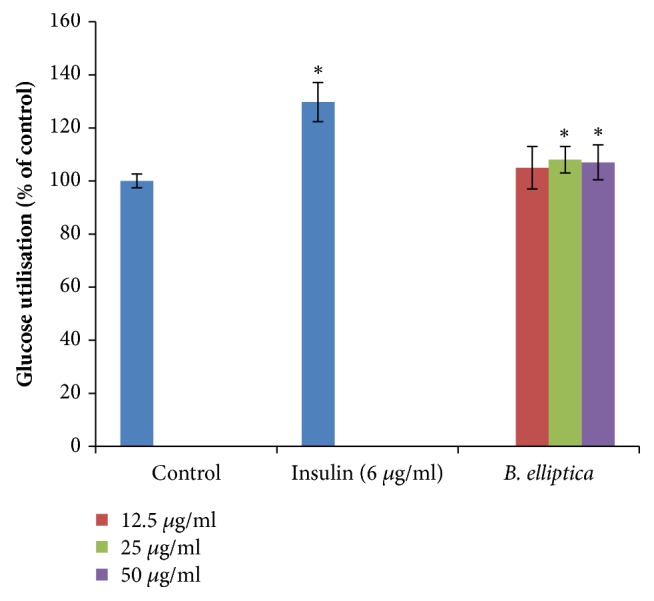
Effect of* B. elliptica* extract on glucose utilization in L6 myoblast. Cells were treated for 48 h in the presence or absence of varying concentration of the plant extract. Data expressed as mean ± SD (n= 4). *∗* indicates a significant increase relative to the untreated control (p < 0.05). No significant increase relative to the positive control (insulin) was noted.

In L6 myoblast cells, the crude extract of* B. elliptica* showed weak potential in lowering blood glucose levels at all the concentration tested (Figure 4). In the majority of cases, the crude extract (108%) also caused a slight increase in glucose uptake in L6 cells but weak at the highest concentration (50 *μ*g/ml) tested when compared to the untreated control (100%). However, insulin (6 *μ*g/ml) used as a positive control, produced better stimulation of glucose uptake in L6 cells with a response of 129.7%.

**Figure 5:**
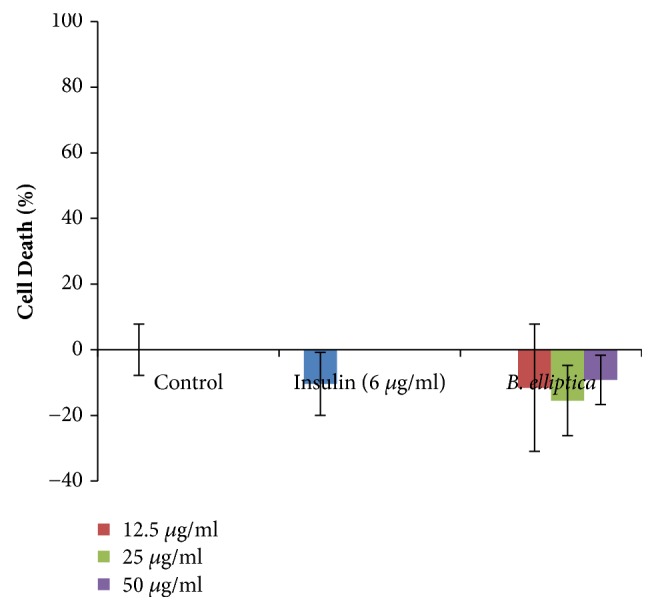
Toxicity of* B. elliptica* extract to L6 myoblast cells used for glucose uptake assay. Data represent the mean ± SD (*n* = 4). No significant increase relative to the untreated control was noted.

The treatments of the extract and insulin in the L6 cell determined by the MTT assay indicated no potential toxicity as shown in Figure 5. It was also observed that the extract and insulin were significantly different from the control thereby proliferating the L6 cells at all the concentration investigated for the glucose uptake.

### 3.4. Lipid Accumulation in 3T3-L1 Preadipocytes

**Figure 6:**
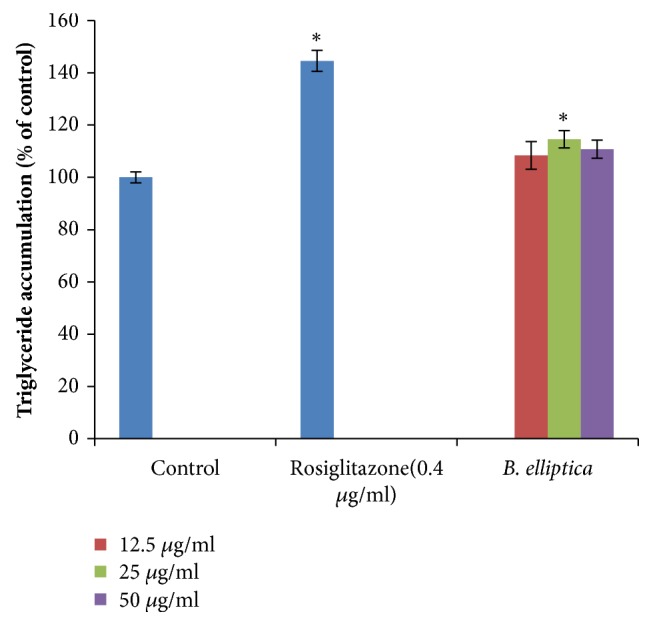
Effect of aqueous extract of* B. elliptica* on triglyceride accumulation in 3T3-L1 preadipocytes. Two days after confluence preadipocytes were treated for an additional two days with varying concentrations of plant extract or rosiglitazone. Data were expressed as mean ± SD (n= 4). *∗* indicates a significant increase relative to the untreated control (control) (p < 0.05). No significant decrease relative to untreated control was noted.

The effect of the* B. elliptica *extract on lipid accumulation in 3T3-L1 preadipocytes is shown in Figure 6. The extract displayed no significant reduction in lipid accumulation in 3T3-L1 cells at all the concentrations (12.5 *μ*g/ml and 25 *μ*g/ml) investigated compared to the untreated control cells. However, the crude extract exhibited an increase in lipid accumulation in 3T3-L1 cells by 110.8% at the highest concentration (50 *μ*g/ml) tested but less marked when compared to the positive control, rosiglitazone (144.6%).

### 3.5. Glucose Metabolism as a Reflection of Insulin Secretion

**Figure 7:**
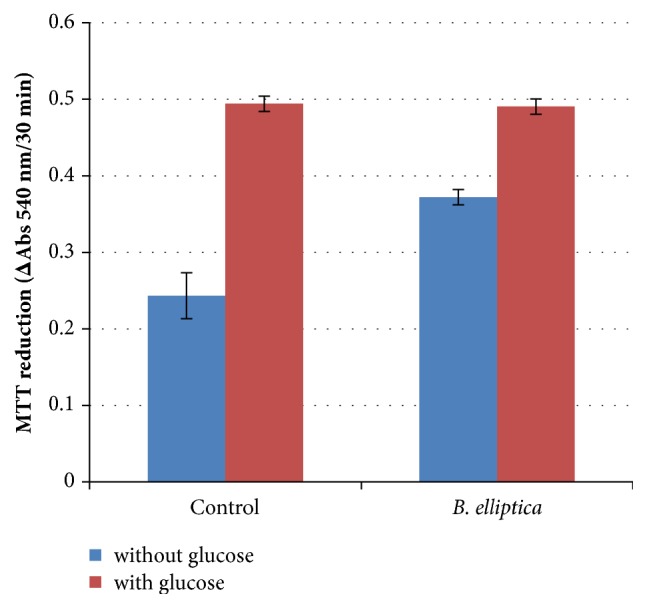
The effect of* B. ilicifolia* on MTT reduction in INS-1 cells. Data are expressed as mean ± SD (n = 6). No significant increase relative to the untreated control (with glucose) was noted.

The effect of the extract of* B. elliptica *on INS-1 cell glucose metabolism as a reflection of insulin secretion is shown in Figure 7. The results obtained revealed that the plant extract in the presence of glucose did not show any significant increase in INS-1 glucose metabolism when compared to the untreated control at 100 *μ*g/ml. Therefore the crude extract of* B. elliptica *did not provide any stimulation of glucose metabolism above the level observed in control cultures incubated with glucose (Figure 7).

### 3.6. INS-1 Proliferation Assay Using ImageXpress Micro XLS Analysis

**Figure 8:**
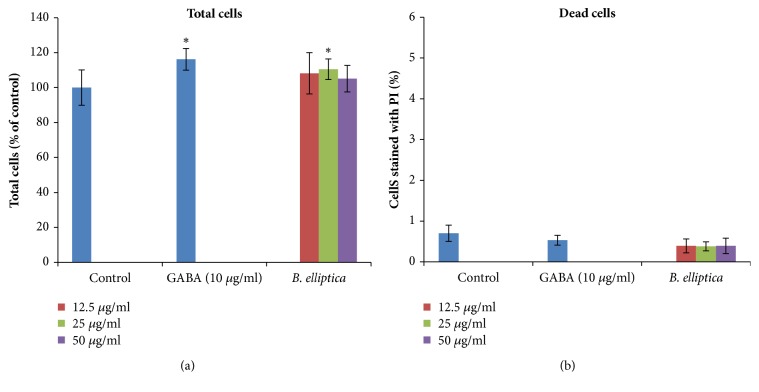
(a) and (b) The effect of aqueous extract of* B. elliptica* on the proliferation of INS-1 beta cells. INS-1 cells were cultured with or without the plant extract for 48 h, and the total cells and dead cells (cells stained with PI) were then determined using the ImageXpress Micro XLS analysis with a Hoechst 33342 dye for total cell counts and propidium iodide for dead cell counts. Data are expressed as mean ± SD (n= 8). *∗* indicates a significant increase relative to the untreated control (p < 0.05).

In this study, the effect of aqueous extract of* B. elliptica* in INS-1 cell was examined using the ImageXpress Micro XLS system (Figures 8(a) and 8(b)). The results indicated that* B. elliptica* extract caused a significant increase in INS-1 cells at 25 *μ*g/ml when compared to the untreated control (Figure 8(a)). However, at the highest concentration (50 *μ*g/ml) tested, no significant increases were seen. It was also observed that the extract not only showed proliferation of INS -1 cells but also showed low levels of dead cell (< 1%) at all the tested concentrations (Figure 8(b)). Nevertheless, neither the extract nor the untreated control evaluated caused a higher significant increase in INS-1 cells than the positive control, GABA (116.2%).

### 3.7. Inhibition of Nitric Oxide Production in RAW Macrophage Cells

**Figure 9:**
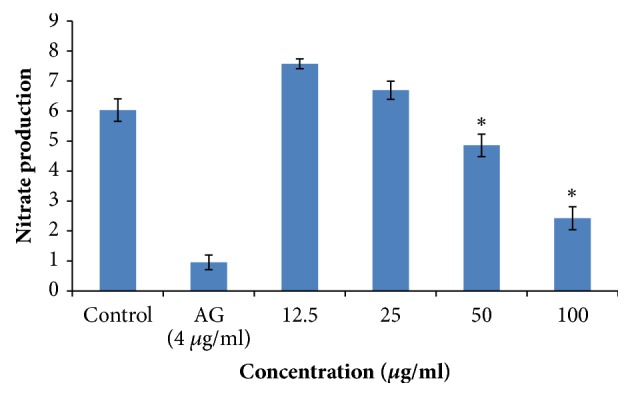
The effect of aqueous extract of* B. elliptica* on NO production by LPS-stimulated RAW macrophage cells. Concurrent MTT assay indicates no significant toxicity under the experimental conditions (data not shown). Data are expressed as mean ± SD (n = 4). *∗* indicates significant decrease relative to the untreated control (p < 0.05). No significant decrease relative to the positive control (aminoguanidine) was noted.

The crude extract of* B. elliptica* significantly inhibited the NO accumulation in LPS activated RAW macrophage cells in a concentration-dependent manner, although the effect was significantly different from that of the positive control, aminoguanidine (0.96 ± 0.2) (Figure 9). In comparison, the extract significantly reduced NO formation at a concentration of 50 *μ*g/ml (4.9 ± 0.3) and 100 *μ*g/ml (2.4± 0.3) when compared to the untreated control (6.0± 0.3). It was also observed that the number of viable activated macrophages cells was not affected by the extract as determined by MTT viability cells assays, thus indicating that an inhibition of the NO production was not due to cell death (data not shown).

### 3.8. Alpha-Amylase and Glucosidase Inhibition Assay

**Figure 10:**
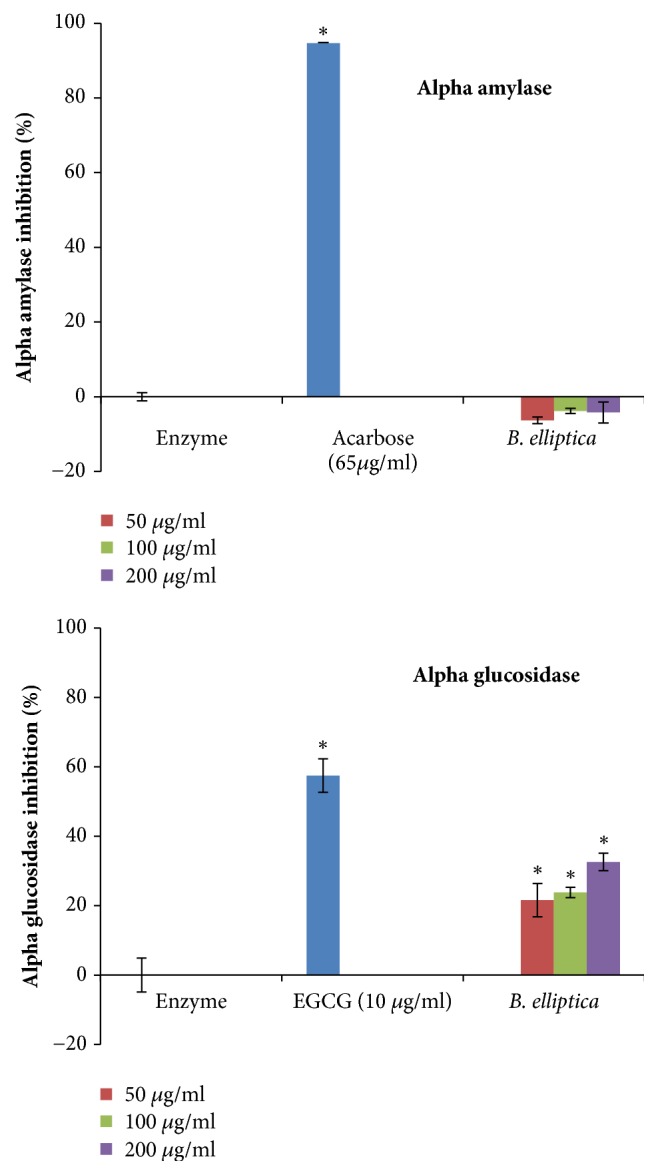
The effect of aqueous extract of* B. elliptica* on alpha-amylase and alpha-glucosidase activity. EGCG: epigallocatechin gallate. Data expressed as mean ± SD (n = 4). *∗* indicates a significant increase relative to the untreated control (enzyme) (p < 0.05). No significant increase relative to the positive controls (acarbose and epigallocatechin gallate) was noted.

In this study, the results indicated that* B. elliptica* extract exhibited no significant effect on alpha-amylase but showed a weak significant effect on alpha-glucosidase at all the tested concentrations (Figure 10). At the highest concentration (200 *μ*g/ml) investigated, the extract displayed appreciable effect on alpha-glucosidase by 32.6%. However, acarbose and EGCG, as positive controls, were far more effective in the respective assays than the extract and untreated control, exhibiting percentage inhibitory activities of 94.7 % and 57.5% against alpha-amylase and alpha-glucosidase, respectively.

### 3.9. Lipase Inhibition Assay

**Figure 11:**
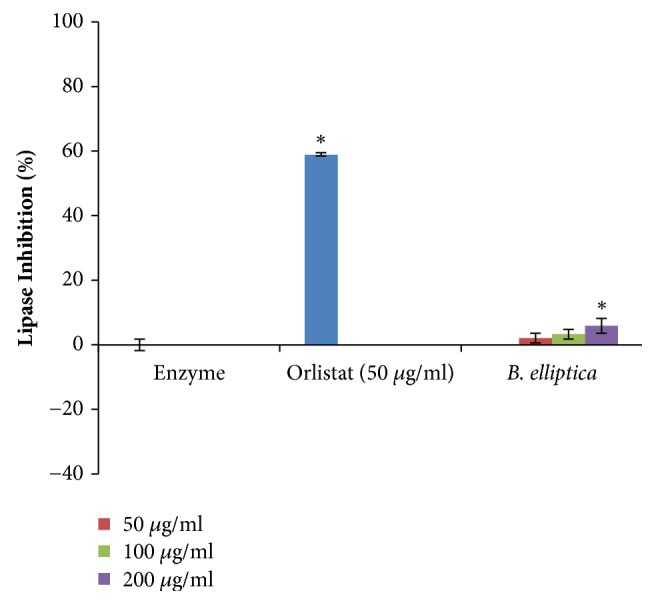
The effect of aqueous extract of* B. elliptica* on pancreatic lipase activity (%). Data expressed as mean ± SD (n = 4). *∗* indicates a significant increase relative to the untreated control (enzyme) (p < 0.05). No significant increase relative to the positive control (orlistat) was noted.

The extract of* B. elliptica* exhibited weak significant inhibition against pancreatic lipase at all the concentration investigated in a concentration-dependent manner (Figure 11). The highest inhibition obtained for* B. elliptica* extract was 5.9% which was much lower when compared to orlistat (58.8%), a known lipase inhibitor. This suggests that the crude extract of* B. elliptica* may not be considered to offer antidiabetic activities via mechanisms related to inhibition of lipase activity

### 3.10. DPP-IV Inhibition Assay

**Figure 12:**
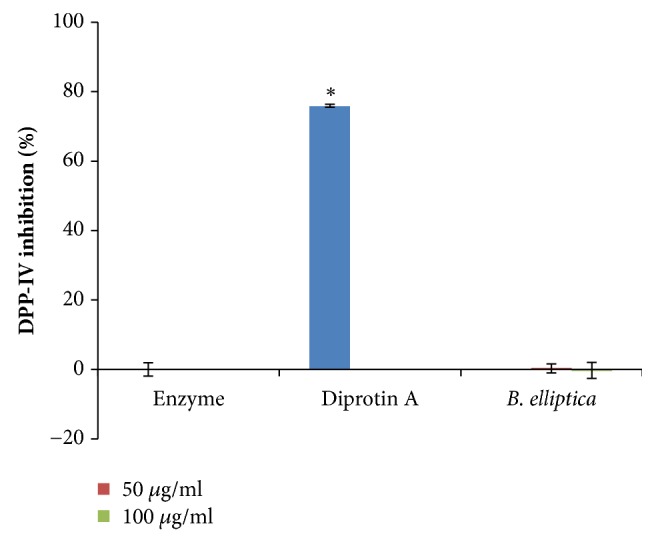
The effect of aqueous extract of* B. elliptica* on DPP-IV activity (%). Data expressed as mean ± SD (n= 4). *∗* indicates a significant increase relative to the untreated control (enzyme) (p < 0.05). No significant increase relative to the positive control (diprotin A) was noted.

The aqueous leaf extract of* B. elliptica *displayed no significant inhibition at all the tested concentrations compared to the positive control (diprotin A) which exhibited 75.8% inhibition of DPP-IV activity (Figure 12). However, there was no significant inhibition of the extract when compared to the untreated control.

### 3.11. Protein Glycation Assay

**Figure 13:**
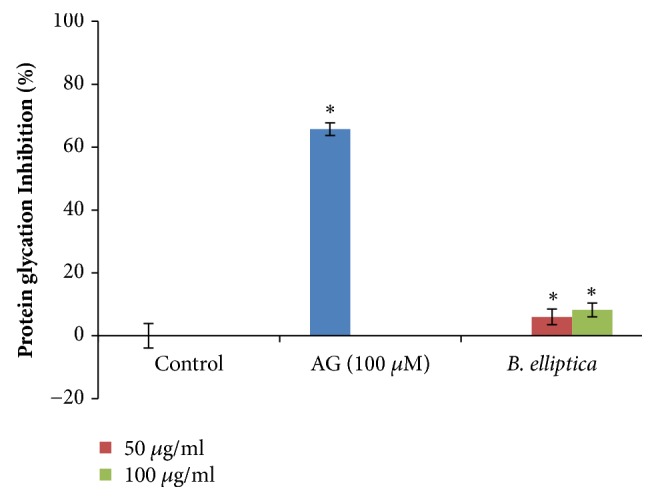
The effect of aqueous extract of* B. elliptica* on protein glycation (%). Data expressed as mean ± SD (n = 4). *∗* indicates significant increase relative to the untreated control (control) (p < 0.05). No significant increase relative to the positive control (aminoguanidine) was noted.

The results revealed that the extract displayed a weak significant inhibition against protein glycation at all the tested concentration in a dose-dependent manner. However, at the highest concentration (100 *μ*g/ml) investigated, the extract inhibited protein glycation by 8.2%, but this was in contrast to aminoguanidine which produced 65.7% inhibition (Figure 13).

### 3.12. Collagenase Inhibition Assay

**Figure 14:**
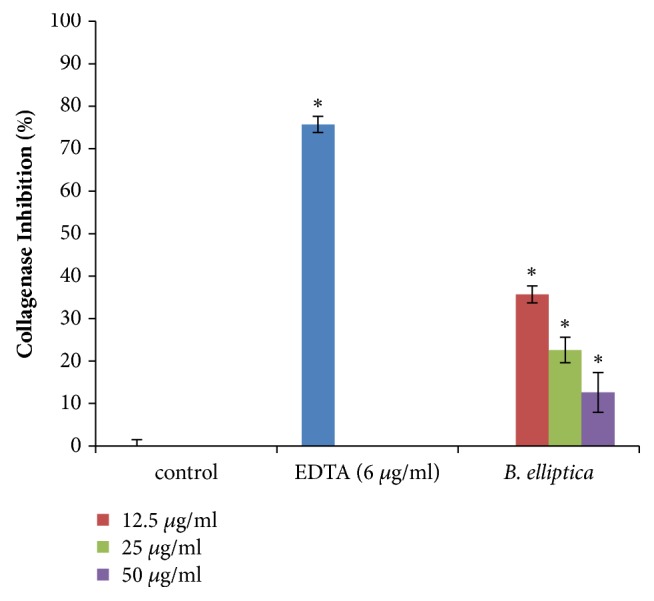
The effect of aqueous extract of* B. elliptica* on collagenase activity (%). Data expressed as mean ± SD (n= 4). *∗* indicates significant increase relative to the untreated control (control) (p < 0.05). No significant increase relative to the positive control (EDTA) was noted.

In this study,* B. elliptica *exhibited a dose-dependent inhibition of collagenase activity with percentage inhibitions of 35.7%, 22.6%, and 12.6% obtained at the concentrations of 12.5 *μ*g/ml, 25*μ*g/ml, and 50 *μ*g/ml, respectively (Figure 14). However, the activity of the extract was actually in contrast with that of the positive control (EDTA) which demonstrated 75.7% inhibition of collagenase activity.

### 3.13. Cytochrome P450 (3A4) Inhibition Assay

**Figure 15:**
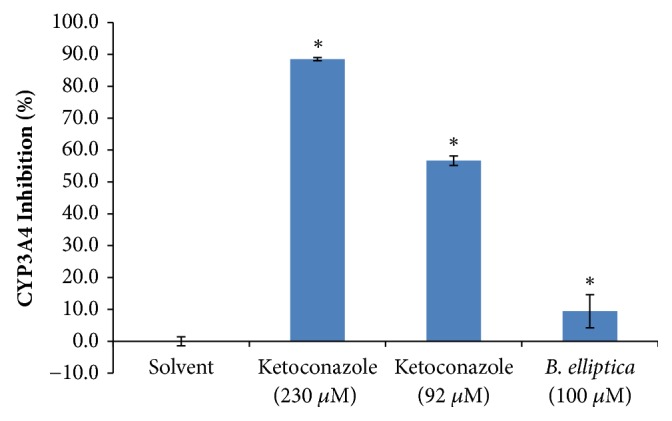
The effect of aqueous extract of* B. elliptica* on CYP3A4 activity (%). Data expressed as mean ± SD (n= 4). *∗* indicates a significant increase relative to the untreated control (solvent) (p < 0.05). No significant increase relative to the positive controls (ketoconazole) was noted.

Figure 15 shows the results of the effect of* B. elliptica* extract against CYP 3A4 enzyme. In this study, a weak significant effect was observed on CYP3A4 enzyme by the extract from* B. elliptica *(9.4 %) at the concentrations investigated (Figure 15). Nevertheless, the plant extract evaluated exhibited a significantly less inhibition of CYP3A4 than the standard positive inhibitor, ketoconazole at 230 *μ*M (88.5 %) and 92 *μ*M (56.7 %).

## 4. Discussion

The identification of toxicity is an important requisite if traditional medicine is to be integrated into public health programmes. In the present study, cytotoxicity effect indicated that less than 50% cell death was recorded for HepG2 cells treated with the extract even at the highest dose of 200 *μ*g/ml. The extract displayed low level of toxicity when compared to other plants used in previous studies that have shown significant toxicity (> 50% cell death) at different concentrations (17 *μ*g/ml, 100 *μ*g/ml, and 200 *μ*g/ml) [18–20]. Therefore the relatively low level of toxicity exhibited by the extract of* B. elliptica* raises prospects that aqueous extract of* B. elliptica *could be potentially safe for the users.

The present study has employed various biochemical and cell-based assays to identify the potential mechanism(s) of probable antidiabetic actions of extract prepared from* B. elliptica*. The result obtained in this study on glucose uptake using HepG2 and L6 cells demonstrated that* B. elliptica* extract increased glucose uptake in HepG2 cells when compared to berberine. However, no significant glucose uptake was observed in L6 cells (skeletal muscles) when compared to the positive control, insulin. This suggests that the crude extract of* B. elliptica*, therefore, mimics metformin by increasing glucose uptake in the liver. Metformin belongs to the category of the biguanide class of oral hypoglycemic. It exerts its hypoglycemic effect through activation of the AMP-activated protein kinase (AMPK) in the liver, which in turn may lead to various pharmacologic effects, including inhibition of glucose, lipid synthesis, and also improved hepatic sensitivity to insulin [21, 22]. The presence of phytochemicals compounds such as phenols, terpenoids flavonoids, and flavanols has been reported to suppress glucose release from the liver and also enhances glucose uptake in hepatic thereby regulating intracellular signalling pathway [23]. Therefore, the glucose uptake observed in HepG2 cells for this extract in this study may be as a result of the presence of polyphenols previously reported in the extract [7]. On this basis, a mechanism of action of* B. elliptica *may be hypothesized which could be linked to activation of the insulin signalling cascade, resulting in stimulation of GLUT 2 that facilitates the translocation of glucose into the cell.

The ability of adipose tissue to accommodate excess lipid can be exceeded in patients suffering from obesity resulting in the abnormal accumulation of lipid in other tissues, such as muscle, liver, and pancreatic islet leading to physiological dysfunction (lipotoxicity) [24]. However, the results obtained for lipid accumulation in 3T3-L1 using Oil red O staining indicated that the extract displayed no significant reduction in lipid accumulation in 3T3-L1 adipocytes suggesting that the extract might not be a good therapeutic agent in lowering lipid profiles.

In pancreatic beta cells, glucose metabolism is very important for glucose stimulated-insulin release. It has also been indicated that stimulation of improved glucose metabolism leading to enhancing ATP production in pancreatic *β* cells could lead to increased secretion of insulin [25]. On the other hand, our results indicated no significant stimulatory effect on the activity of enzymes involved in glucose metabolism in INS-1 cells, therefore, suggesting that* B. elliptica* extract does not enhance glucose stimulated-insulin release and might not be a good therapeutic agent in stimulating insulin secretion.

Several lines of studies have indicated that the decline in pancreatic beta-cell mass, through either an increase in apoptosis or a decrease in proliferation, is believed to be one of the major contributory factors in the development of type 2 diabetes [26, 27]. Proinflammatory cytokines and/or abnormally high blood glucose promote the development of oxidative stress in pancreatic islets to aggravate beta-cell death by apoptosis [26]. In this study, the treatment of INS-1 cells with* B. elliptica *extract at 25 *μ*g/ml caused a significant increase in INS-1 cells. However, it could be deduced from this study that the aqueous leaf extract of* B. elliptica *may possess a mitogenic effect or able to induce the expression of growth stimulating factors such as insulin-like growth factors (IGF-1), growth hormone (GH), and prolactin, which have been reported to stimulate or enhance beta-cell proliferation [28]. This is the first study to examine the effect of aqueous extract of* B. elliptica *in INS-1 cell death and proliferation. However, aqueous and methanol extracts of* Brachylaena discolor *have been reported to possess a proliferative effect on Hela cell lines [29] which supported the findings obtained in this study. This result gives an indication that the aqueous leaf extract of* B. elliptica *has a proliferative or anti-apoptotic effect or both and could benefit patients suffering from diabetes.

Nitric oxide has been reported to contribute to the pathogenesis of diabetes [30]. Our result on inhibition of nitric oxide production in RAW macrophage cells indicated that* B. elliptica *extract reduced NO production in RAW macrophage cells. This reduction of nitric oxide production could be probably through inhibition of the expression of iNOS. Consequently, iNOS needs to be regulated in order to prevent excessive production of NO during inflammation. However, the observed result exhibited by the extract of this plant further confirms the potential antioxidant activity reported from a previous study [7]. Several lines of studies have also indicated that polyphenolics play a major role in inhibition of several inflammatory facilitators including nitric oxide [31, 32]. Therefore, it could be deduced from this study that the observed NO inhibitory effect of this plant could be attributed to the presence of polyphenolics such as phenols and flavonoids in the extract.

Presently, there are several antidiabetic drugs used to treat or manage diabetes and the mechanisms of action of these drugs are well known. These include the inhibition of alpha-amylase, alpha-glucosidase, lipase, and DPP-IV enzyme. Our results indicated that the extract demonstrated no significant inhibition on alpha-amylase, glucosidase, lipase, and DPP-IV when compared to the respective positive controls. However, the mild inhibition on alpha-amylase observed in this study by the extract is probably not physiologically relevant. This suggests that the antidiabetic mechanism of* B. elliptica *is therefore not through the inhibition of these enzymes.

Studies have shown that one of the consequences of abnormal high blood glucose in patients suffering from diabetes is protein glycation [33]. Protein glycation is the reaction between reducing sugar (galactose, mannose, glucose, fructose, and ribose) and free amino group of a protein reversibly leading to the formation of adducts (Schiff and Amadori products) and over a long period produces glycation products. These reactions play a significant role in the development of pathogenesis of diabetic complication [34]. The extract displayed weak significant inhibition on protein glycation and collagenase at the concentration investigated compared to aminoguanidine and EDTA. However, this study represents the first attempt to investigate both protein glycation and anticollagenase properties of the extract of* B. elliptica.* Therefore, it can be deduced from this study that the aqueous leaf extract of* B. elliptica* might not be a good therapeutic agent in alleviating diabetic complications.

Herbs contain potent inhibitors of one or more CYP450 enzyme subtypes (CYP1A2, CYP2A6, CYP2E1, and CYP3A4) causing potential herb-drug interactions with diverse effect [35]. The extract showed weak inhibition of CYP3A4 at the concentration tested, indicating that extract may be taken concurrently with antidiabetic drugs which are activated or metabolized by cytochrome P450 with a low risk of pharmacokinetic interaction. The result of this study corroborated with previous studies who reported weak inhibition of some medicinal plants on CYP3A4 enzyme [36, 37]. Studies have also indicated that phytochemicals such as alkaloids and saponins have been reported to inhibit various CYP450s subtypes [35, 38]. Therefore, the weak inhibition on CYP3A4 observed in this study could be attributed to the low content of alkaloids and saponins in the extract [7].

## 5. Conclusions

In summary, our findings suggest that* B. elliptica* exert its hypoglycemic activity independent of insulin and through restoring or maintaining the health and proper functioning of the beta-cell and the pancreas. The possible mechanisms of antidiabetic action of* B. elliptica* may be linked to strong proliferative and antioxidative effects and interactions with insulin receptors, leading to activation of the MAPK and P13K pathways, which results in the translocation of glucose transporters. This observed antidiabetic activity of this extract might be due to their phytochemical constituents previously reported [7]. The findings from this study therefore support the folkloric usage of* B. elliptica* in the treatment of diabetes. However, due to the potential toxicity of the plant extract it must be prescribed with caution. The results observed in this study also support the use of this plant with other antidiabetic drugs that are metabolized by this isoform of cytochrome P450.

## Data Availability

The data used to support the findings of this study are available from the corresponding author upon request.
